# Prevalence of Internet Gaming Disorder and Its Impact on Routine Activities Among Dental Students in Belagavi, India: A Cross-Sectional Study

**DOI:** 10.7759/cureus.65315

**Published:** 2024-07-24

**Authors:** Prajakta Chavan, Anil Ankola, Roopali Sankeshwari, Atrey Pai Khot, Anu Sara Varghese, Varkey Nadakkavukaran Santhosh, Deepika Bhatt

**Affiliations:** 1 Public Health Dentistry, KLE Vishwanath Katti Institute of Dental Sciences, KLE Academy of Higher Education and Research, Belagavi, IND; 2 Public Health Dentistry, Goa Dental College and Hospital, Panjim, IND; 3 Public Health Dentistry, King George's Medical University, Lucknow, IND; 4 Public Health Sciences, KLE Vishwanath Katti Institute of Dental Sciences, KLE Academy of Higher Education and Research, Belagavi, IND

**Keywords:** behavioural addiction, gaming addiction, smartphone addiction, internet addiction, gaming disorder

## Abstract

Background: Internet gaming has gained popularity since the millennium, greatly expanding both the game industry and the player base. Moreover, internet gaming disorder (IGD) is a condition included in the most recent version of the Diagnostic and Statistical Manual of Mental Disorders 5 (DSM-5) for further study.

Aim: To assess the prevalence of IGD and its association with physical symptoms among dental students in Belagavi, India.

Materials and methods: A cross-sectional study was conducted among 385 dental students, chosen through simple random sampling. Data were gathered using a pre-validated, closed-ended questionnaire, incorporating the 9-item DSM-5 short version. The questionnaire was administered to undergraduate students during theory classes and collected after 10 minutes, while postgraduate students received it in their respective departments. To identify significant differences, Chi-square and analysis of variance (ANOVA) tests were applied, with statistical significance established at p ≤ 0.05.

Results: A total of 385 responses were collected, comprising 86 males (22.3%) and 299 females (77.7%). Among the respondents, 28 (7.2%) were diagnosed with IGD, while 123 (31.8%) were identified as risky gamers. Significant differences were found between disordered, risky, and normal gamers concerning sleep-related problems (p ≤ 0.05).

Conclusion: The prevalence of IGD and risky gamers was 7.2% and 31.8% respectively. There was a significant association between disordered, risky, and normal gamers with daily routine activities.

## Introduction

One of the most popular online pastimes is playing games on the internet. Unfortunately, losing control over online video gameplay has led to a number of unfavourable effects. Owing to its significant negative effects on mental health, the problematic gaming behaviour was named internet gaming disorder (IGD) and included in section III of the Diagnostic and Statistical Manual of Mental Disorders 5 (DSM-5). According to DSM-5, IGD is defined as “persistent and recurrent use of the Internet to engage in games, often with other players, leading to clinically significant impairment or distress” [1]. Globally, 3.05% of people have gaming disorders, which is equivalent to various substance use disorders (2.6%) and obsessive-compulsive disorders (0.6% to 3.5%) [2]. The prevalence rates in Asian countries were found to be higher than those in other areas, with East Asia being among the highest worldwide [3].

In India, the total prevalence of IGD was estimated to be 3.50% among school children, with a higher prevalence in males (8.8%) than in females (0.8%) [4]. India had around 365 million internet gamers in the year 2020. By 2022, this number had increased to 510 million. India ranked first in total app store downloads of online games [5].

According to the research, computer game addiction affects a number of health conditions, including psychosocial problems like anxiety and depression as well as physical problems including dry eye symptoms, musculoskeletal pain, and near-miss accidents [6]. Prolonged video game play can strain the visual system, which in some people can result in headaches, light-headedness, nausea, and vomiting [7]. Excessive mobile gaming has been found in the past to have a negative impact on sleep, leading to increased sleep onset latency and sleep deficit [8]. Additionally, long-term players may have musculoskeletal issues, with neck pain being the most typical [9].

Due to the ban on outdoor activities, public gatherings, and social interaction, the coronavirus disease 2019 (COVID-19) pandemic has had a substantial impact on many elements of human life globally [10,11]. The World Health Organization (WHO) emphasized that increased screen and gaming may emerge during the COVID-19 pandemic. This will eventually increase the likelihood of internet and gaming addiction [12]. Supporting these predictions, it is reported that during this pandemic, the prevalence of IGD has grown by 1.6 times [13]. Therefore, reinstating the prevalence and relevant variables of IGD has become crucial.

Dental students often experience high levels of stress due to the demanding nature of their academic and clinical responsibilities. Common stressors include academic pressure, clinical workload, fear of making mistakes, time management issues, and financial concerns [14]. The learning environment for dental students is quite challenging since the field involves both academic and practical expertise, as well as hands-on training and regular patient contact [15]. Usually, people who are under stress frequently feel inadequate, guilty, and inclined to hold themselves responsible for their shortcomings [16]. The cycle of self-blame, tension, and anxiety can then be fuelled by emotions of helplessness and hopelessness, which can ultimately result in unhealthy coping mechanisms, such as compulsive gaming [17].

There is a dearth of research on the prevalence of disordered gaming and its detrimental effects on daily activities and dental students' physical health, despite the fact that many studies have been conducted to look into the potentially dangerous effects of IGD and its effects on daily activities, including those of medical professionals. In light of this, the current study set out to determine the prevalence of IGD among dentistry students in the city of Belagavi as well as how this disorder affected their everyday lives.

## Materials and methods

The current cross-sectional study follows the Strengthening the Reporting of Observational Studies in Epidemiology (STROBE) reporting requirements. It was conducted among dental students in the Belagavi city of Karnataka state. Ethical approval was obtained from the Institutional Research and Ethics Committee (reference no.: 1536). The study was conducted in the year 2022 from March to June. The participants were informed of the study's objectives, and their written informed consent was obtained. All dental college participants who were willing to provide informed consent for the study were included.

A pilot study was carried out on a representative sample of 10 students to detect any design flaws such as word ambiguity, inability to understand the questions, and other questionnaire-related errors. The questionnaire's reliability was deduced to be 0.85 using Cronbach's alpha, and its validity was deduced using face validity (84%) and content validity ratio (78%). A valid questionnaire was created after the questionnaire was further refined based on pre-test feedback to make it more pertinent and specific to the objectives of the study.

Sample size estimation and sampling technique

Simple random sampling was used for the study. A list of dental students was obtained from the administrative office and the participants were selected using a table of random numbers. The sample size estimation was done using the formula based on a study conducted by Bhaskar et al. in 2022 [18], where prevalence was taken to be 17% and 27% and α error of 0.05 and a power of 0.90. A total of 391 responses were received and 385 were taken for the final analysis after excluding the unfilled questionnaire.

Questionnaire details

The questionnaire was self-administered and comprised 24 close-ended questions in the English language (see Appendices). The questionnaire was sectioned into three parts. The first part included total questions related to demographic details and the details of gaming activities. The second was a series of nine questions as a scale meant to diagnose the participants for IGD. According to the DSM-5, when a person fits five (or more) of the nine criteria over the course of a year, they can be diagnosed with IGD [19]. Every item on both IGD scales was preceded by the phrase "During the previous 12 months..." according to this temporal norm, and the questionnaire was based on nine criteria: “preoccupation”, “tolerance”, “withdrawal”, “persistence”, “escape”, “problems”, “deception”, “displacement”, and “conflict” [20]. Questions to evaluate daily activities such as study, slumber, and appetite were included in the third section. Physical symptoms were assessed in terms of posture and musculoskeletal problems.

Diagnosis and categorization of the participants

The DSM-5 notes that gaming must cause "significant impairment or distress" in numerous areas of a person's life [1]. To assess that, five of the nine diagnosis criteria (preoccupation or obsession, withdrawal, tolerance, loss of control, loss of interest, continued overuse, deceiving, escape of negative feelings, functional impairment) are proposed. According to these criteria, the participants were diagnosed as disordered and non-disordered. Furthermore, according to the criteria given by Lemmens et al. [20], participants having a score >1 can be diagnosed as risky gamers. Hereafter, the participants were divided into disordered gamers, risky gamers, and non-gamers.

Statistical analysis

Collected data were entered in MS Excel (Microsoft® Corp., Redmond, WA, USA) and analysed using the Statistical Package for the Social Sciences (IBM SPSS Statistics for Windows, IBM Corp., Version 21.0, Armonk, NY). The Shapiro-Wilk test was used to evaluate the normality of the data distribution, and the data was found to be normally distributed. Descriptive statistics were applied for the frequency distribution and percentage of students. The Chi-square test was applied to check for the association between the physical symptoms and the diagnosis. Consecutively, the analysis of variance (ANOVA) test was applied to test significance among categories of diagnosis. The statistical significance was set at p ≤ 0.05 for all the tests.

## Results

This study aimed to determine the prevalence of IGD and its association with physical symptoms and routine activities among dental students in Belagavi city. The sociodemographic characteristics of the participants are presented in Table 1. There were 385 responses, with 299 (77.7%) female and 86 (22.3%) male participants. The majority of the participants were final-year students (15.4%), followed by postgraduates (15%), with first-year students being the least represented (7.2%). Among the participants, 28 were diagnosed as disordered gamers according to DSM-5 criteria, while the majority (61%) were non-gamers. Additionally, 123 participants were classified as risky gamers.

**Table 1:** Sociodemographic details of the participants All values are expressed as the frequency with percentages (in parentheses).

Characteristics	Frequency (%) (n=385)
Age	
Below 20	114 (20.9)
21-22	144 (26.4)
23-24	53 (9.7)
25 and above	74 (13.6)
Sex	
Male	86 (22.3)
Female	299 (77.7)
Year	
Postgraduate	80 (14.7)
Intern	59 (10.8)
Final Year	84 (15.4)
Third Year	61 (11.2)
Second Year	62 (11.4)
First Year	39 (7.2)
Prevalence of Internet Gaming Disorder (IGD)	
Non-gamers	234 (61)
Risky gamers	123 (31.8)
Disordered gamers	28 (7.2)

Table 2 depicts the descriptive statistics of the questionnaire. Of the participants, 253 (65.7%) played video games, with 83 (21%) preferring online games, 51 (9.4%) preferring offline games, and 125 (22.9%) preferring both. Nearly 56% of participants reported playing video games for one to three hours daily.

**Table 2:** Descriptive statistics of the questions asked All values are expressed as the frequency with percentages (in parentheses).

S. No.	Questions	Response	Frequency (%)
1	Do you play video games?	Yes	253 (65.7)
		No	132 (34.2)
2	What kind of video games do you prefer?	Online games	83 (21.5)
		Offline games	51 (13.2)
		Both	116 (30.1)
		Do not play games	132 (34.2)
3	How many hours do you spend daily gaming?	1-3 h	56 (10.3)
		<1 h	184 (33.8)
		>3 h	13 (2.4)
5	Has there been any time when all you could think of was only playing video games?	Yes	79 (14.5)
		No	306 (56.1)
6	Have you felt unsatisfied because you wanted to play more?	Yes	63 (11.6)
		No	322 (59.1)
7	Have you been feeling miserable when you were unable to play a video game?	Yes	37 (6.8)
		No	348 (63.9)
8	Were you unable to reduce your time playing games, after others had repeatedly told you to play less?	Yes	106 (19.4)
		No	279 (51.2)
9	Have you played games so that you would not have to think about annoying things?	Yes	156 (28.6)
		No	229 (42.0)
10	Have you had arguments with others about the consequences of your gaming behaviour?	Yes	43 (7.9)
		No	342 (62.8)
11	Have you hidden the time you spend on video games from others?	Yes	38 (7.0)
		No	347 (63.7)
12	Have you lost interest in hobbies or other activities because gaming is all you wanted to do?	Yes	30 (5.5)
		No	355 (65.1)
13	Have you experienced serious conflicts with family, friends or partner because of video gaming?	Yes	33 (6.1)
		No	352 (64.6)

Association between physical symptoms and the diagnosis of IGD

Physical symptoms like headache, neck pain, tearing, eyestrain, and pain in the wrist and interphalangeal joints were assessed among the participants. Chi-square analysis between the physical symptoms and the diagnosis showed a very significant association (p < 0.001) (Table 3). The most particular physical complaint experienced among the disordered gamers category was headache (4.6%) followed by eyestrain (2.8%) and neck pain (2.3%). The most common symptom in the risky gamers category was eyestrain (13.1) and 12% of risky gamers showed no symptoms.

**Table 3:** Comparison of various physical symptoms between disordered gamers, non-gamers, and risky gamers All values are expressed as the frequency with percentages (in parentheses). * indicates statistically significant (p≤0.01), ¥ indicates statistical test used Chi-square.

S. No.	Symptoms	Diagnosis			p-value
		Disordered Gamers	Non-gamers	Risky Gamers	
1	Headache^¥^	18 (4.6)	52 (13.4)	44 (11.3)	0.000*
2	Neck pain^¥^	9 (2.3)	35 (9.0)	29 (7.5)	0.002*
3	Excessive blinking^¥^	3 (0.8)	4 (1.0)	8 (2.1)	0.022
4	Tearing^¥^	4 (1.0)	10 (2.6)	20 (5.1)	0.000*
5	Eyestrain^¥^	11 (2.8)	45 (11.6)	51 (13.1)	0.000*
6	Pain in wrist and interphalangeal joints^¥^	5 (1.3)	11 (2.8)	10 (2.6)	0.019
7	No symptom^¥^	9 (2.3)	150 (38.6)	50 (12.9)	0.000*

Comparison of various routine activities with the diagnosis of IGD

The participants' daily routine activities were evaluated in the following areas: physical symptoms, sleep, appetite, and others. The three diagnostic categories, disordered gamers, risky gamers, and non-gamers, were compared using ANOVA. There was a statistically significant difference (p < 0.001) between these groups related to problems viz. appetite and physical symptoms (Table 4).

**Table 4:** Comparison of physical symptoms, sleep, appetite, and other problems between disordered gamers, non-gamers, and risky gamers All values are expressed as the frequency with percentages (in parentheses). ** indicates an analysis of variance (ANOVA) test used. * indicates statistically significant (p≤0.01).

S. No.	Area	Options	Categorization of participants n (%)			Total n (%)	F-value	p-value
			Disordered	Non-gamers	Risky Gamers			
Physical symptoms**								
1	How much does this pain interfere with your normal work or studies?	Not at all	8(2.1)	169(43.8)	55(14.2)	233(42.8)	18.09	0.000*
		A little bit	8(2.1)	42(10.9)	49(12.7)	99(18.2)		
		Moderately	7(1.8)	14(3.6)	13(3.4)	34(6.2)		
		Quite a bit	5(1.3)	5(1.3)	6(1.6)	16(2.9)		
		Extremely	0	3(0.8)	0	3(0.6)		
2	Do you feel your posture has changed due to prolonged video gaming?	Not at all	7(1.8)	201(52.1)	72(18.7)	280(51.4)	44.81	0.000*
		A little bit	10(2.6)	27(7.0)	31(8.0)	68(12.5)		
		Moderately	5(1.3)	4(1.0)	9(2.3)	18(3.3)		
		Quite a bit	4(1.0)	2(0.5)	9(2.3)	15(2.8)		
		Extremely	2(0.5)	0	2(0.5)	4(0.7)		
Sleep**								
3	How many hours of sleep do you get?	4-6hrs	10(2.6)	48(12.5)	24(6.2)	82(15.0)	2.09	0.124
		6-8 hrs	17(4.4)	165(42.9)	87(22.6)	269(49.4)		
		>8 hrs	1(0.3)	21(5.5)	12(3.1)	34(6.2)		
4	How would you rate your sleep quality overall?	Very good	4(1.0)	81(21.0)	36(9.4)	121(22.2)	3.48	0.032*
		Fairly good	19(4.9)	136(35.3)	75(19.5)	230(42.2)		
		Fairly bad	4(1.0)	13(3.4)	11(2.9)	28(5.1)		
		Very bad	1(0.3)	4(1.0)	1(0.3)	6(1.1)		
Appetite**								
5	Do you play video games while eating?	Very often	2(0.5)	5(1.3)	2(0.5)	9(1.7)	14.55	0.000*
		Sometimes	7(1.8)	17(4.4)	21(5.5)	45(8.3)		
		Less Often	9(2.3)	13(3.4)	13(3.4)	35(6.4)		
		Never	10(2.6)	199(51.7)	87(22.6)	296(54.3)		
6	Do you cut down your eating/meal time in order to play video games?	Very often	5(1.3)	2(0.5)	4(1.0)	11(2.0)	31.09	0.000*
		Sometimes	5(1.3)	4(1.0)	5(1.3)	14(2.6)		
		Less Often	3(0.8)	4(1.0)	7(1.8)	14(2.6)		
		Never	15(3.9)	224(58.2)	107(27.8)	346(63.5)		
7	Do you experience loss of appetite due to prolonged hours of gaming?	Very often	2(0.5)	2(0.5)	4(1.0)	8(1.5)	14	0.000*
		Sometimes	3(0.8)	6(1.6)	6(1.6)	15(2.8)		
		Less Often	6(1.6)	4(1.0)	14(3.6)	24(4.4)		
		Never	17(4.4)	222(57.7)	99(25.7)	338(62.0)		
Others**								
8	Do you cut down the amount of time spent on studies or work in order to play video games?	Very often	7(1.8)	7(1.8)	11(2.9)	32 (5.9)	33.37	0.000*
		Sometimes	12(3.1)	12(3.1)	30(7.8)	68 (12.5)		
		Less Often	4(1.0)	4(1.0)	49(12.7)	89 (16.3)		
		Never	5(1.3)	5(1.3)	32(8.3)	196 (36.0)		
9	Do you experience any accidents or falls while walking due to being engrossed in playing?	Very often	7(1.8)	3(0.8)	3(0.8)	13(2.4)	36.28	0.000*
		Sometimes	3(0.8)	6(1.6)	5(1.3)	14(2.6)		
		Less Often	5(1.3)	2(0.5)	9(2.3)	16(2.9)		
		Never	13(3.4)	223(57.9)	106(27.5)	342(62.8)		

## Discussion

The history of gaming addiction began in 1983 with the publication of the first study that claimed video game addiction is a concern among students [21]. Ever since both the quantity and quality of studies concerning internet gaming addiction have increased [22]. Furthermore, DSM-5 classified problematic gaming addiction as IGD in the year 2013 [1]. Since the turn of the millennium, internet gaming has grown in popularity, significantly boosting both the gaming market and the player base. Moreover, India, with one of the largest and youngest populations in the world, has emerged as a key market for video games [23].

The present study was conducted to examine the prevalence of this emerging disorder, its association with physical symptoms, and its impact on routine activities. The results showed that gaming was a common activity among dental students, with more than half of the participants (65%) reporting playing games regularly. However, a small proportion of the total sample (7.2%) met at least five of the nine criteria needed for the DSM-5 diagnosis of IGD and were categorized as disordered gamers. This aligns with the overall prevalence of 0.7-27.5% IGD from different countries according to a systematic review by Mihara and Higuchi [24].

In a study by Shrestha et al. in 2020, around 69.2% of the students stated that the stress of COVID-19 had led to an increase in their gaming behaviour. Additionally, 86.5% of the participants said that they prefer playing games on the internet as it helps them cope with the pressure of the current circumstances [25]. A similar study was conducted by Balhara et al. where it was found that the majority of the participants (50.8%) reported engaging in more games during the lockdown. They also expressed a stronger agreement than students who did not enhance their gaming habit with the notion that gaming assisted in reducing stress associated with the COVID-19 epidemic and following public health measures like lockdown, quarantine, and social isolation [26].

Player Unknown's Battlegrounds (PUBG) was one of the most popular games in India in recent years before it was banned, along with other Chinese apps, according to a case series released in 2021 by Mamun et al. [27], the detrimental consequences of PUBG included exam failure, running away from home, hospitalization, accidentally drinking acid instead of water, suicide, and even death. In the present study, the most played games among dental students were Among Us, followed by Ludo King and PUBG.

In the present study, there was a significant association between IGD and physical symptoms. A study conducted by Baskar among medical students showed a similar considerable association between online gaming and physical problems among gamers [18]. Of the 802 participants in that study, 47% reported experiencing physical issues like headaches, neck pain, and pain in the hands and wrists attributed to gaming. Similarly, in the present study, disordered gamers complained of headaches and other eye-related symptoms like eye strain, tearing, and excessive blinking compared to risky and non-gamers which aligns with a study by Mowatt et al., on the prevalence of computer vision syndrome wherein eye strain, dry eyes, double vision and blurred vision were common symptoms among medical students [28].

A cross-sectional study by Wong et al. found a positive correlation between IGD and sleep quality. It was found that continuous gaming may lead to physical discomfort, such as muscular pain and headache [29]. However, in the current study, the majority of individuals who identified as disordered or risky gamers reported getting six to eight hours of adequate sleep. This might be attributable to the fact that sleep issues became most severe when gaming lasted more than an hour every day, whereas in the current study, the majority of individuals who played games played for less than an hour each day [30].

This study has some drawbacks, the primary being the smaller sample size. Furthermore, the variables for checking physical symptoms and association with routine activities are self-reported and may be subject to inaccurate memories, leading to bias. Despite these limitations, to our knowledge, this study is the first to investigate IGD among dental students. To better understand the harmful implications of gaming disorder, more longitudinal studies with large samples of the general population are required.

## Conclusions

The prevalence of IGD is increasing dramatically, which might have long-term harmful repercussions on both dedication to study and the health of the students. The current study found the prevalence of IGD and risky gamers was 7.2% and 31.8% respectively. There was a significant association between disordered, risky, and normal gamers with daily routine activities. This indicates that prompt professional attention is required in controlling this rising fret. Additionally, there is a need to increase awareness of the condition.

## Appendices

Questionnaire

NAME:                                                                                              Age:

Sex:   1. Male                                   2. Female

You Are a:   1. Post Graduate               2. Intern                      3. Final Year UG     

                          4. Third Year UG                      5. Second Year UG                

  (For Question No. 3, 5, 16 multiple options can be selected)

1.     Do you play video games?

                   i.          Yes

                 ii.           No

2.     What kind of video games do you prefer?

                      i.     Do not play games

                    ii.     Online games

                  iii.     Offline games

                  iv.     Both

3.     On which gaming system do you spend your maximum time?

                      i.     Do not play games

                    ii.     Mobile

                  iii.     PC/Laptop

                  iv.     PlayStation

4.     How many hours do you spend daily gaming?

                      i.     Do not play games

                    ii.     1-3 h

                  iii.     <1 h

                  iv.     >3 h

5.     Which video games you are playing? (Tick your option)                                                             

                  i.          PUBG (BGMI)                                                            

                ii.           Valorant                                                                      

               iii.          Call of Duty                                                                 

               iv.          Far cry                                                                         

                 v.          Counterstrike                                                              

               vi.          Ludo King                                                                   

             vii.           Among Us                                                                   

             viii.          Rocket League

               ix.          War zone

                 x.          Mobile legends

               xi.          League of legends

              xii.           FIFA

             xiii.          Need for Speed

             xiv.          GTA

              xv.          Others                                                                             

6.     Do you cut down the amount of time spent on studies or work in order to play

           video games?

                      i.     Very often

                    ii.     Sometimes

                  iii.     Less Often

                  iv.     Never

State whether yes/no

During the last year ….

7.     Has there been any time when all you could think of was only playing video games?

8.     Have you felt unsatisfied because you wanted to play more?

9.     Have you been feeling miserable when you were unable to play a video game?

10.  Were you unable to reduce your time playing games, after others had repeatedly told you to play less?

11.  Have you played games so that you would not have to think about annoying things?

12.  Have you had arguments with others about the consequences of your gaming behavior?

13.  Have you hidden the time you spend on video games from others?

14.  Have you lost interest in hobbies or other activities because gaming is all you wanted to do?

15.     Have you experienced serious conflicts with family, friends, or partners because of video gaming?

16.  Do you experience any of the following physical symptoms? (Tick your options)

       i.          Headaches

     ii.          Neck pain

   iii.          Excessive blinking

   iv.          Tearing

     v.          Eyestrain

   vi.          Pain in wrist and interphalangeal joints

  vii.          No symptom

17.  How much does this pain interfere with your normal work or studies?

                   i.     Not at all

                 ii.      A little bit

                iii.     Moderately

                iv.     Quite a bit

                  v.     Extremely

18.  Do you feel your posture has changed due to prolonged video gaming?

                   i.     Not at all

                 ii.      A little bit

                iii.     Moderately

                iv.     Quite a bit

                  v.     Extremely

19.  How many hours of sleep do you get?

                   i.     4-6hrs

                 ii.      6-8 hrs.

                iii.     >8 hrs.

20.  How would you rate your sleep quality overall?

                   i.     Very good

                 ii.      Fairly good

                iii.     Fairly bad

                iv.     Very bad

21.  Do you play video games while eating?

                   i.     Very often

                 ii.      Sometimes

                iii.     Less Often

                iv.     Never

22.  Do you cut down your eating/meal time to play video games?

                   i.     Very often

                 ii.      Sometimes

                iii.     Less Often

                iv.     Never

23.  Do you experience loss of appetite due to prolonged hours of gaming?

                   i.     Very often

                 ii.      Sometimes

                iii.     Less Often

                iv.     Never

24.  Do you experience any accidents or falls while walking due to being engrossed

           in playing?

                   i.     Very often

                 ii.      Sometimes

                iii.     Less Often

                iv.     Never
